# The Laboratory Method in Dental Education

**Published:** 1894-12

**Authors:** Edward C. Kirk

**Affiliations:** Philadelphia


### THE LABORATORY METHOD IN DENTAL EDUCATION.[1]

BY EDWARD C. KIRK, D.D.S., PHILADELPHIA.

Probably in no other period of a similar extent, in the history of dentistry, has more active progress in all its departments been made than in the past fifteen years. The greatest advances in dental pathology and bacteriology have been among the fruits of dental progress of this period, giving us the solution of the etiology of dental caries, and possibly of that other *bête noire* of the dental practitioner, pyorrhœa alveolaris. This period has also witnessed the renaissance of dental mechanical art as manifested in modern crown- and bridge-work, with its increased and more accurate knowledge of the physical and chemical properties of the metals and alloys employed in the work. There has been a no less important advance in the therapeutic treatment of pulpless teeth, and a similar increase in our knowledge of the uses of filling-materials, especially in the domain of plastics, with a corresponding improvement in their composition. To the same period belongs also the application of cocaine as a local anæsthetic, with its marked impress upon various operative procedures; the introduction of hydrogen dioxide as an oral and dental antiseptic, as well as of numerous other and valuable therapeutic agents, each of which has added its quota towards the betterment of dental methods. As

---

[1] Read before the American Academy of Dental Science, Boston, May 2, 1894.

a necessary and correlated factor of this energetic advance in dental science and art, there has been a well-marked activity along the channels of dental educational effort. In the matter of dental educational methods the quality of the product is as with more material processes, largely governed by the forces of public requirement. A dental graduate, discovering that the educational equipment furnished him by his Alma Mater is inadequate to enable him to successfully meet the requirements of actual practice, brings his influence to bear upon the sources of dental training with a view to their improvement to the extent of correcting the defects which he has recognized in them. Hence it is that a wholesome attrition is maintained on the one hand between the profession, through individual effort, in its periodical literature, and by means of its representative State examining boards, and the dental educational institutions on the other, whereby there results a constant and steady improvement in our educational system. The most notable improvements in this respect have been the lengthening of the term of pupilage and the broadening of the curriculum to include a greater range of topics.

These advances are of acknowledged importance and value. They were so palpably needed and so generally admitted to be necessary that no argument is required to enforce the wisdom of their adoption. There is, however, another advance step which has already been taken by a few colleges, regarding which the same unanimity is not evident,—viz., a radical departure from the old didactic and class method of imparting instruction, and the substitution in its place of the laboratory and individual method of teaching. It is this latter method that I shall ask you to consider with me this evening. The question involved is one of pedagogics, and embraces principles which are by no means new, the only element of novelty in the case being the adaptation of certain well-known educational principles to the special requirements of the dental student. All methods of imparting instruction may be broadly classified under two principal systems,—viz., those wherein the student receives his knowledge at first hand by direct sense impressions derived from material objects or phenomena, and those wherein he acquires his knowledge second-hand from mental impressions conveyed orally by the teacher. Fortunately for our purpose, the history of modern dentistry is comprised practically within the allotted limits of a human life, and it is therefore an easy matter to find among its practitioners to-day men whose educational equipment will represent the result of each of the various systems that have in the course of time been tried. In its earlier history, before the establishment of dental schools, the apprentice system was the only means by which the dental student could gain a knowledge of his calling. Under this system, while it was necessarily varied by individual circumstances, and the amount of instruction imparted depended exclusively upon the ability and the intelligence of the teacher, still the system had one most excellent feature in that it brought the student into close personal relations with his instructor,—made him a witness of, as well as a participant in, the operative procedures of the office, and an active agent in all the dental laboratory work. Under the apprentice system the student listened to no lectures filled with technicalities which he could not understand; nor was the practical part of his instruction confided to demonstrators of recent graduation and little experience. What teaching he received may have been limited as to breadth and extent, but as far as it went it was thorough, for it was obtained first hand and directly from the objects and phenomena with which he was called upon to deal. As evidence that the old apprentice system bore good fruit, it is only necessary to recall to your minds the army of pioneer practitioners who by their ability and wisdom laid broadly and well the foundations upon which the fair structure of modern dentistry has been reared. The demand for a more liberal education for the dentist brought into existence the dental college, which, following the example of its medical ancestor, introduced the lecture system, with its accompaniment of large classes, its amphitheatre, and professorial oratory, to all of which the student submitted as to the inevitable, and, after securing his diploma, gained a large part of the actual working knowledge of dental procedures—which he failed to obtain in college—on the unfortunate patients who consulted him during the first years of his practice. It is true that the mechanical laboratory and operative clinic were designed to supply to the student his needed practical training. This in a measure they do, but as usually conducted the system is handicapped by two serious defects, the first of which is the lack of trained and competent teachers, whose entire time and energy are utilized for educational ends; and, second, the lack of a graded system of laboratory teaching adapted to the rate of educational progress which the average student should make. Until recently, the usual system, if it can be so termed, was for the student to have assigned to him a patient, the care of whose case he attempted to carry out under the supervision of the demonstrator, quite regardless of the nature of the treatment needed and the student's competency to proceed with it. I distinctly recall that during the first day of my attendance at college I was assigned a patient for the extraction of a molar tooth, and, having had no instruction upon the subject, in fact, never having seen the operation performed, I was inclined to rebel; but upon the assurance of the demonstrator that there was no other way to learn, I made the attempt, and succeeded in breaking off the tooth level with the gum, leaving a throbbing, pulsating fragment of pulp exposed in its centre. My subsequent attempt to remove the root failed, because, in my awkwardness, the cold beak of the forceps came in contact with the exposed pulp, giving the patient such a sudden shock of pain that he jumped from the chair and incontinently fled. My experience in putting in my first gold filling was quite analogous to my extracting case, as I managed to drill into the pulp in making my anchorages. I, of course, learned after a time to avoid these disastrous features in my work, but if a proper system of instruction had at that time been in vogue, they could have been avoided from the beginning. My own experience, as indicated by the incidents related, was by no means unusual, and all present can no doubt recall similar ones as having occurred during their college career. A system of dental education which permits the green student to acquire his training upon patients, even those of the dental clinic, is inherently bad and little short of malpractice. But quite apart from the moral and humanitarian questions which it involves, such a system is defective, because it does not admit of graded instruction and training, progressing from the simple to the more complex procedures. The knowledge so gained is likely to be imperfect because of this and lacking in homogeneity.

The modelling of the dental course upon the classical pattern furnished us by the medical colleges, while it has in some ways been productive of good results, has at the same time burdened us with a certain lack of originality and a feeling of dependence that have hampered progress in dental educational methods. Recently, dental teachers seem to be awakening to the fact that the operative procedures of dentistry demand especial and more careful consideration than heretofore; and that better methods are needed for the training of students in the practical manipulative phase of their work. It is being felt more and more strongly that the training required to properly fit a man for the practice of dentistry is a peculiar one, and requires a curriculum adapted to that end. It has come to be recognized that no amount of didactic instruction can impart manipulative skill or dexterity to the student; and that it is as much a function of the college course to furnish this element as it is to impart intellectual training. It has been found by careful test and observation that intelligent laboratory training means for the student something more than dexterity and manipulative skill. Wherever it has been applied as an educational means, manual training has resulted in an increased intellectual status, a brain cultivation fully the equivalent of the manual dexterity which is incident to it. In fact, the mental development which in nearly all cases has been seen to occur as a corollary of the manual training method has led a number of practical educators to regard the specific manual training factor as merely incidental to its possibilities as a developer of the intellect; the training of the hand, as it were, being regarded as a means solely for the cultivation of the brain. The importance of this fact and its demonstrated value as an educational means have led to its systematic introduction as one of the factors in the public school system of this country. In several countries of Europe it has already been in operation for some years. Dental educators are coming to recognize the value of the facts as noted, and there is a growing tendency to adapt and utilize them in the training of dental students. Hence it is that the laboratory and clinic-room are beginning to take on a more important significance. Instead of being regarded as workshops, where the student is to put into practice the instruction he has received in lectures, they are becoming the arenas where instruction is given in the presence of the case, and the didactic phase is being correspondingly modified to deal with the scientific principles involved, and the methods of study to be pursued in the laboratory and clinic. The laboratory method of teaching may be taken to include all methods which bring the student into contact with objects or phenomena, in such a way that he can acquire a knowledge of them through or by means of the impressions which they make upon his several senses. Its intrinsic superiority over the didactic method is easily seen, when we consider that in the one case the student is the observer, and receives his impressions directly from the object or phenomenon, while in the other his concept is produced through an association of ideas conveyed by the words of his teacher, who may or may not have been an original observer. The distinction here made is not by any means new; in fact, it would be difficult to name its originator, but it has repeatedly received new impetus, so far as its practical application is concerned, by the work of a number of philosophic thinkers, who, recognizing its importance as a factor in educational processes, have elaborated it in connection with various channels of intellectual progress. Educational experiments, with the remarkable results achieved in special cases by Pestalozzi, and the writings of Rousseau are in evidence of the value of this method of instruction. The application of the method by Seguin to the training of idiots was a peculiar demonstration of its merit. A more familiar example is furnished by the work of Friedrich Froebel, the originator of the kindergarten system, which is founded upon the same principle. Or again, the laboratory method, as applied in the teaching of all departments of natural and physical science, of which anatomy, physiology, bacteriology, pathology, etc., are to us familiar examples, will show the practical working of the principle under consideration. These again furnish all the precedent that may be required for its adaptation to the needs of the dental student. In 1890, I called attention to this subject in a paper on "The Manual-Training Idea in Dental Education;" emphasizing its importance, not only because of the rational physiological basis of the method, but because under such a system the facts are directly impressed upon the mind of the student with at least a minimum, if any, of mental effort upon his part, and the impressions so made are relatively permanent. No definite plan was proposed for the utilization of the method in dental instruction, the discussion of the psychological side of the subject being the point at issue. About this time or previous to it, Dr. G. V. Black had elaborated and put into successful operation a plan of instruction for his students, adapted to both mechanical and operative dentistry. These he has termed "Technic Courses," and they are based wholly upon the laboratory method. I have had the opportunity of witnessing the practical workings of the method in at least two dental colleges, and any one who has had the same opportunity will, I am quite sure, pardon my enthusiasm in speaking of it. It is but just to its originator, and those who are engaged in developing it, to state that the technic system of dental instruction is still in its formative stage, and that it is being carefully and gradually, though steadily, elaborated, as experience leads to a clearer knowledge of how best to apply it to the mutual advantage of student and instructor. Under the technic system the untrained freshman is not permitted to gain his experience and manual dexterity upon the clinical patient, as under the old system, but enters upon a practical laboratory study of the organs which he will afterwards be called upon to treat in the mouth. He is made familiar with the morphology of the several classes of teeth by actual study of extracted specimens; he is taught to select, classify, arrange, and articulate them in their proper order and relationship; he studies their structure and the arrangement of their several tissues by dissecting them, just as he does the anatomy of the body in general, which is only properly learned by dissection. The system of dental dissection pursued consists in making sections of the teeth in the vertical axis both laterally and antero-posteriorly; also cross-sections through three or more planes of the crown and root. As each section is made, a record of it is taken by inking the cut surface of the tooth on an ink pad, and then pressing it on paper, which gives a printed silhouette representation of the section as cut. In crown sections a line between the enamel cap and dentine, at the position of the stratum granulosum, is cut out by means of a graver, and a similar line is cut between the cement and the dentine of the root. An impression from a section so made gives a fair though rough definition of the various structures of the tooth as severally related. Besides the knowledge gained by the student as to the relations of the tooth-tissues, and especially of the size, conformation, and general relations of the pulp-chamber, he has by this time gained a very fair knowledge of the texture and physical properties of the several dental tissues, and a definite amount of manipulative skill in cutting them. This is further enhanced by an extension of his study, by the same methods, to the treatment and filling of cavities. An assortment of carious teeth having a variety of cavities is furnished to the student; after he has properly arranged and mounted these in a suitable support, he is taught to prepare, treat, and fill them. He is instructed in the proper use of chisels, excavators, nerve instruments, pluggers, etc.; he studies the lines of enamel cleavage, the formation of anchorages, and how to prepare his cavities upon correct mechanical principles, and is familiarized with the relations of cavities to the pulp-chamber. He becomes acquainted with the physical properties of his filling-materials and their proper manipulation. He is taught to look for and recognize pulp exposure, and what to do in that event. He practically treats his "dummy patient" for all the dental diseases which ordinarily occur,—caries, pulp-capping, pulp-devitalization, alveolar abscess, pyorrhœa, etc. Of all his work he makes a permanent record, which is examined, passed upon, and proper credit given therefor by his instructor. All of this, let it not be forgotten, has to be gone through with before the student is permitted to operate for a clinical patient. Is it not evident that the possibilities of such a system are, beyond all comparison, superior to the plan of turning a class of untrained students into a clinic-room to shift for themselves, with an occasional suggestion from an overworked demonstrator?

The same general method of training is carried out with respect to the department of prosthetic dentistry. The student pursues a course of practical laboratory work, starting out with the simplest and most elementary work, which gives him a familiarity with the materials with which he has to deal. From this he proceeds by degrees to the more complex operations that depend for their successful issue upon his practical knowledge of the principles involved in the assemblage and proper co-ordination of the simpler facts which he has previously learned. The technic system, or, as I prefer for the present to term it, the laboratory method, is being extended to other departments of dental instruction besides those noted. I have recently received from a prominent teacher the syllabus of his course on dental metallurgy, which is based on the same idea. Chemistry, which at one time was taught almost exclusively by the laboratory method, until there arose a tendency to indulge to extremes in the didactic and theoretical method of teaching, has more recently tended to revert to its proper environment, the chemical laboratory; so that the student is now being taught more of the actual facts of the science, and is less likely to be asphyxiated by a flood of formulæ and chemical mathematics than formerly. The same may be perhaps asserted of electricity, if one may take as an indication a statement constituting the preface of an important work on the subject, that "there are but two kinds of electricity, one found on college black-boards, and the other found in nature; this book treats of the latter."

Nature is the best instructor, and it is a closer contact with nature which we need in the study of dentistry; the function of dental educators, in fact, all educators, being, as the term really implies, to serve as intelligent guides in the interpretation of the phenomena which nature in her own way presents for our study and enlightenment. The technic system of dental instruction has its opponents as do all advances of human intelligence, but the criticisms which have been made upon it are those respecting its details rather than of its governing fundamental principles. These latter require no defence, as when fully comprehended they become self-evident truths. Their adaptation, so far as dentistry is concerned, is but recent, and as the initial stages of such movements in all cases require subsequent modification, so necessarily will the technic system, as experience shall more clearly indicate its shortcomings. What we need, then, in view of the growing popularity of the laboratory system, is to clearly understand the principles upon which it is founded, and to develop it in its detailed application to dental education, so that its highest possibilities for good may be realized. This, in the first place, means the education and training of teachers along the lines indicated. Professors may be had for the asking, but expert teachers are a rarity. We need less of the amphitheatre, with its oratory and rhetoric, and more of class-room, laboratory, and seminar work conducted in contact with dental material, by the aid of syllabus, paper and pencil, instruments, and appliances, all under the guidance of *teachers of dentistry.* The geniuses in science and art are those who, without exception, have received their instruction from nature and her phenomena, as manifested in their several departments. They have, by giving intelligent heed to her teachings, learned, each in his degree, to understand her language and interpret it to others. Because of their ability to translate something of the unknown into terms of the known, the world has called them great. It is the broad principle contained in this truth, which I ask you to consider in relation to the education of men preparing for the pursuit of our calling, to the end that her standard-bearers of the future may be worthy of their high trust.

